# Drinking patterns and the distribution of alcohol-related harms in Ireland: evidence for the prevention paradox

**DOI:** 10.1186/s12889-019-7666-4

**Published:** 2019-10-22

**Authors:** Claire O’Dwyer, Deirdre Mongan, Seán R. Millar, Marion Rackard, Brian Galvin, Jean Long, Joe Barry

**Affiliations:** 1 Health Research Board, Grattan House, 67–72 Lower Mount Street, Dublin 2, Ireland; 2 School of Public Health, University College Cork, Fourth Floor, Western Gateway Building, Cork, Ireland; 3 HSE Alcohol Programme, 2nd Floor Block D, Parkgate Business Centre, Parkgate Street, Dublin 8, Ireland; 4 Department of Public Health and Primary Care, Institute of Population Health, 6th Floor, Russell Centre, Trinity College Dublin, Dublin, 24 Ireland

**Keywords:** Alcohol, Drinking patterns, Harm, Population studies, Prevention paradox, Ireland

## Abstract

**Background:**

According to the prevention paradox, the majority of alcohol-related harms in the population occur among low-to-moderate risk drinkers, simply because they are more numerous in the population, although high-risk drinkers have a higher individual risk of experiencing alcohol-related harms. In this study we explored the prevention paradox in the Irish population by comparing alcohol-dependent drinkers (high-risk) to low-risk drinkers and non-dependent drinkers who engage in heavy episodic drinking (HED).

**Methods:**

Data were generated from the 2013 National Alcohol Diary Survey (NADS), a nationally representative cross-sectional survey of Irish adults aged 18–75. Data were available for 4338 drinkers. Respondents dependent on alcohol (as measured by DSM-IV criteria), respondents who engaged in monthly HED or occasional HED (1–11 times a year) and low-risk drinkers were compared for distribution of eight alcohol-related harms.

**Results:**

Respondents who were dependent on alcohol had a greater individual risk of experiencing each harm (*p* < .0001). The majority of the harms in the population were accounted for by drinkers who were not dependent on alcohol. Together, monthly and occasional HED drinkers accounted for 62% of all drinkers, consumed 70% of alcohol and accounted for 59% of alcohol-related harms.

**Conclusions:**

Our results indicate that the majority of alcohol consumption and related harms in the Irish population are accounted for by low- and moderate-risk drinkers, and specifically by those who engage in heavy episodic drinking. A population-based approach to reducing alcohol-related harm is most appropriate in the Irish context. Immediate implementation of the measures in the Public Health (Alcohol) Act (2018) is necessary to reduce alcohol-related harm in Ireland.

## Introduction

Alcohol use is the seventh leading risk factor for both deaths and disability-adjusted life years globally and is the leading risk factor among those aged 15–49 [1]. Recent research indicates that there is no level of consumption at which alcohol is not harmful to health [1]. Social harms arising from alcohol consumption, such as damage to work and home life, may also constitute a significant burden to drinkers [2–4]. Ireland has one of the highest per-capita alcohol consumption rates in the European Union, with alcohol consumption and hazardous drinking patterns in Ireland projected to increase over the next decade [5].

The selection of an appropriate target population represents a significant challenge to the design and implementation of polices aimed at reducing alcohol consumption and related harms. Two different, although not mutually exclusive, policy approaches are generally taken in order to reduce alcohol-related harm [4, 6]. A high-risk approach targets a small number of high-risk drinkers, while a population-based strategy aims to reduce alcohol-related harms through interventions targeted at the whole population. Lobbyists representing the alcohol industry often argue that policies to reduce alcohol-related harm should be based exclusively on the former, high-risk approach [7]. However, Krietman [8] and subsequent researchers [6, 9–12] have demonstrated that the majority of alcohol-related harms tend to occur among low and moderate-risk drinkers, simply because they are more numerous in the population than high-risk drinkers, who still have a higher individual risk of experiencing harms. This phenomenon is known as the ‘prevention paradox’ [8].

The prevention paradox has been found to hold true across a range of populations [12–16] when risk is defined in terms of volume of alcohol consumed. However, alcohol-related harms are not only related to volume of alcohol consumed, but also to patterns of drinking, and specifically occasions of heavy episodic drinking (HED) [4, 12–14], typically defined as consuming more than 60 g of pure alcohol in a single sitting [17]. Studies have demonstrated that when occasions of HED are taken into account, the prevention paradox is either weakened or disappears entirely [9–11, 13, 14]. These findings imply that most alcohol-related harms are due to periods of acute intoxication, and because these occasions are most numerous among low and moderate risk drinkers, they account for the majority of alcohol-related harms. This has been referred to as the “second-order” prevention paradox [6, 14, 15].

The distribution of alcohol consumption and harms may play out in a unique way in the Irish context, given the high HED, high volume pattern of consumption [18]. Ireland’s per capita consumption is sixth highest among 36 Organisation for Economic Co-operation and Development (OECD) countries [19]. Findings from a 2013 national household survey on alcohol consumption indicated that 75% of alcohol in Ireland is consumed as part of an occasion of HED [20]. The Public Health (Alcohol) Act (2018) [21] has recently been passed and will introduce a number of population-based strategies to reduce alcohol consumption in Ireland. However, many of these measures have yet to be enacted. Determining the distribution of harms across drinking patterns in Ireland is important in order to establish if a population-based approach is best-suited to reducing alcohol-related harms.

Therefore, the primary aim of the study was to identify patterns of alcohol consumption in Ireland, and how alcohol-related harms are distributed across the population in relation to these drinking patterns. A secondary aim was to investigate whether the prevention paradox is supported in an Irish context when high-risk drinkers are defined as those who meet the criteria for alcohol dependence.

## Methods

### Sampling and study population

Data were generated from the National Alcohol Diary Survey (NADS) [20]. The NADS was a nationally representative cross-sectional survey of a stratified clustered sample of 5991 individuals aged 18–75 years living in private households in Ireland. The sampling frame used for the study was the An Post/Ordnance Survey Ireland GeoDirectory database, a list of all addresses in the Republic of Ireland, distinguishing between commercial and residential dwellings. A multi-stage probability approach to sampling was used. The first stage involved the selection of geographical areas. The second stage involved stratifying the sample according to social class and degree of urbanity to ensure that selected geographical locations were representative of the population. All households selected through this sampling process were visited during the fieldwork period and all adults aged 18–75 years in each household were invited to participate.

Respondents completed a face-to-face interview and self-completed questionnaire. Interviews were administered in participants’ own homes by professional social interviewers via a Computer Assisted Personal Interview (CAPI). Interviews were completed between July and October 2013; the household response rate was 67.2% and the within household response rate was 77.1%. The survey was granted ethical approved by the Royal College of Physicians of Ireland and all participants gave written informed consent for their data to be used for research purposes.

### Measures

#### Number of standard drinks consumed in last week

Over two-thirds (69.1%, *n* = 2997) of those who consumed alcohol in the last 12 months also consumed alcohol in the week prior to the survey. Participants were asked to report all alcohol consumed in the week prior to survey. A standard drink in Ireland contains 10 g of pure alcohol and is equivalent to half a pint of beer, a single pub measure of spirits (35 ml), or a small (100 ml) glass of wine. Respondents were provided with beverage-specific flash cards so they could accurately report how many standard drinks they consumed.

#### Heavy episodic drinking

For the purpose of this study, heavy episodic drinking (HED) was defined as consuming 60 g or more of pure alcohol in a single drinking occasion [17]. Respondents were asked how frequently they consumed six or more standard drinks on a single occasion and given the following response options: “*everyday”, “5–6 times a week”, “4 times a week”, “3 times a week”, “twice a week”, “once a week”, “2–3 times a month”, “once a month”, “6–11 times a year”, “2–5 times a year”, “once in the last 12 months”* and *“never”*.

#### Alcohol dependence

Alcohol dependence was defined according to DSM-IV (Diagnostic and Statistical Manual of Mental Disorders, 4th Edition) criteria, and was measured via self-completed questionnaire using the ten items that denote alcohol dependence from the Composite International Diagnostic Interview [22]. Alcohol dependence was established from a positive response in three or more of the seven domains on the DSM-IV diagnostic criteria in the 12 months before the interview. Only participants who had complete information on both the DSM-IV and on frequency of heavy episodic drinking (HED) were included in the analyses (*n* = 4338).

#### Classification of drinkers

Current drinkers were defined as those who had consumed alcohol on at least one occasion in the last year. Non-drinkers (*n* = 1236, 20.6%), defined as those who had not consumed any alcohol in the last 12 months, were excluded from the analyses. Drinkers were placed into one of the four categories below based on frequency of HED and whether they met the criteria for alcohol dependence:

##### Alcohol dependence

Respondents who met the DSM-IV criteria for alcohol dependence were classified as dependent drinkers (*n* = 299, 6.9%).

##### Monthly HED

Respondents who reported consuming six or more standard drinks at least once a month and who did not meet the DSM-IV criteria for dependence were classified as monthly HED drinkers (*n* = 1368, 31.5%).

##### Occasional HED

Respondents who did not meet the DSM-IV criteria for alcohol dependence and reported consuming 6 or more standard drinks between 1 and 11 times in the previous year were classified as occasional HED drinkers (*n* = 1326, 30.6%).

##### Low-risk drinkers

Drinkers who did not meet the criteria for dependence and who had not engaged in HED on any occasion in the last year were defined as low-risk drinkers (*n* = 1345, 31.0%).

#### Alcohol-related harms

Questions on eight alcohol-related harms were included in the survey. These questions were based on the “adverse social consequences of own alcohol use” from the Standardized Measurement of Alcohol-Related Troubles (SMART), a set of guidelines for the standardisation of drinking population surveys in Europe [23]. Respondents were given the response options of “*yes, once” “yes, more than once”* and *“no”* as to whether they had experienced each of the harms in the last 12 months. The eight harms covered were harm to finances, harm to health, harm to work or study, harm to friendships or social life, harm to home life or marriage, been in a physical fight, been in an accident, and stopped by the police. E.g., “*Have you experienced harms to your finances in the last 12 months due to your own drinking?”*

#### Calculation of total number of alcohol-related harms

The total number of harms from drinking experienced by respondents in the survey was estimated by creating a 16-point scale based on the eight alcohol-related harm questions. Participants were assigned a score of 0 (“*never”*) 1 (“*yes, once”*) or 2 (“*yes, more than once”*) on each of the eight questions depending on their response. Scores on each of the eight harm questions were summed to give each participant a total score of harms that ranged from 0 to 16. Respondents’ scores were summed to create a total number of harms across the survey sample. This score is likely to be an under-estimation of harms, as “*more than once*” could equate to a number greater than 2. However, the purpose of obtaining the total harm experienced by the survey population was not to provide a precise estimate of the number of alcohol-related harms in the population but was to estimate how the harms were distributed across each drinker type. Hence the scoring of the scale in this way was appropriate to the aim of the study.

### Statistical analyses

Statistical analyses were carried out using Stata version 15 (Stata Corporation, College Station, TX, USA) for Windows. Data were weighted with respect to age, gender and regional distribution to ensure they were nationally representative. With consideration for missing values, only valid percentages are reported. Descriptive statistics were used to describe the median (inter-quartile range) and total number of standard drinks consumed by respondents. Associations between socio-demographic variables and drinker type were analysed by cross-tabulations and significance was assessed by the Pearson χ^2^ test. Socio-demographic variables analysed were age, gender, employment, marital status and income. Associations between drinker type and each of the harms experienced from own drinking were analysed by cross-tabulations, using the Pearson χ^2^ test to test for statistical significance. For all analyses an alpha level of *p* < .05 was considered to be statistically significant.

## Results

### Characteristics of current drinkers by drinker type

The bivariate associations between socio-demographic characteristics and drinker-type are presented in Table 1. Of the 4338 respondents included in the analyses, there was a relatively even breakdown of low-risk (31.0%), occasional HED (30.6%), and monthly HED (31.5%) drinkers. Dependent drinkers constituted 6.9% of all drinkers in the study. A higher proportion of males were classified as monthly HED drinkers and dependent drinkers (*p* < .0001). The proportion of monthly HED drinkers and dependent drinkers were highest among the two youngest age groups (*p* < .0001). Just over 70% of dependent drinkers were aged between 18 and 34. Being a student was associated with engaging in HED and being dependent on alcohol (p < .0001). A similar proportion of high-income and low-income earners were reported to be occasional HED or monthly HED drinkers. However, there was a significantly higher proportion of low-income earners (62.0%) compared to high-income earners (38.0%) in the alcohol-dependent group. There was a significant association (*p* < .0001) between being dependent on alcohol or engaging in HED and age of first alcohol use. Over 80% of those meeting the criteria for alcohol dependence, and over 70% of monthly HED drinkers had consumed alcohol before the age of 18.

**Table 1:** Bivariate associations between socio-demographic characteristics and drinker type

	n	Total (*n* = 4338; 100.0%)	Low risk (*n* = 1345; 31.0%)	Occasional HED (*n* = 1326; 30.6%)	Monthly HED (*n* = 1368, 31.5%)	Dependent (*n* = 299, 6.9%)	p
Gender							
Male	2163	49.9%	29.6%	48.8%	67.0%	67.4%	<.0001
Female	2175	50.1%	70.4%	51.3%	33.0%	32.6%	
Age							
18–24	598	13.8%	5.4%	11.6%	20.7%	29.5%	<.0001
25–34	1069	24.7%	17.6%	25.5%	27.1%	41.2%	
35–49	1411	32.5%	32.7%	38.8%	29.8%	16.8%	
50–64	899	20.7%	27.5%	19.1%	18.0%	9.7%	
65–75	359	8.3%	16.7%	5.0%	4.5%	2.8%	
Marital status							
Single/never married	1282	29.6%	15.8%	26.6%	39.9%	57.5%	<.0001
Married/cohabiting	2772	63.9%	74.7%	67.8%	55.8%	35.6%	
Divorced/separated/ widowed	283	6.5%	9.6%	5.6%	4.3%	6.9%	
Employment status							
Employed	2573	59.3%	51.4%	63.7%	63.8%	54.8%	<.0001
Unemployed	399	9.2%	6.3%	8.4%	11.1%	17.0%	
Economically inactive	932	21.5%	37.0%	18.8%	12.0%	7.3%	
Student	433	10.0%	5.3%	9.1%	13.1%	20.9%	
Income							
Under 20,000	2152	55.3%	60.9%	50.7%	52.8%	62.0%	<.0001
Above 20,000	1742	44.7%	39.1%	49.3%	47.2%	38.0%	
Age of first use							
15 or under	808	18.7%	7.7%	18.8%	25.3%	37.5%	<.0001
16–17	1692	39.2%	27.1%	43.5%	45.5%	45.0%	
18–20	1199	27.7%	36.6%	27.2%	22.6%	13.9%	
21+	623	14.4%	28.6%	10.5%	6.6%	3.6%	

*HED: Heavy episodic drinking; Occasional HED; engaged in HED 1–11 times in the last year; Monthly HED; engaged in HED at least once a month in last year; Dependent: meets criteria for DSM-IV alcohol dependence*

### Association between drinker type and harms from own drinking

Overall 29% (*n* = 1206) of drinkers experienced at least one harm from their own drinking in the last year. As seen in Table 2, experiencing harm to finances was the most frequently reported harm (19.3%) followed by harm to health (15.8%). Being in an accident (6.8%) and being stopped by the police (6.2%) were the least frequently reported harms. There was a linear increase between type of drinker and the frequency of reporting each of the eight harms. For all eight harms, dependent drinkers reported the highest frequency, followed by monthly HED drinkers, occasional HED drinkers and finally, low risk drinkers.

**Fig. 1:**
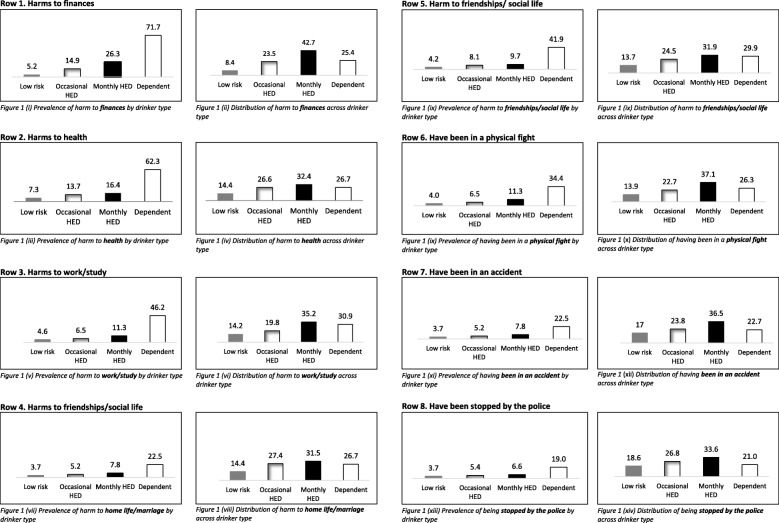
displays the prevalence and distribution of alcohol-related harms across each of the four types of drinker in the study. The figures on the left hand side illustrate the percentage of each drinker type that experienced each of the alcohol-related harms in the last 12 months. The figures on the right-hand side depict the overall proportion of each of the harms that were accounted for by each type of drinker

The “*yes, once*” and “*yes, more than once*” response options were combined to display the frequency of drinkers who had reported experiencing that harm at least once in the last 12 months. Figure 1 illustrates the linear association between drinker type and experiencing each of the eight harms, with the proportion of harms lowest among low-risk drinkers (3.7–7.3%) and highest among those who were alcohol dependent (19.0–71.7%). The proportion of harms experienced by low-risk drinkers, occasional HED drinkers, and monthly HED drinkers was relatively low, with proportions increasing substantially among those dependent on alcohol (Fig. 1).

**Table 2:** Harms experienced from own drinking by drinker type

Weighted count	n	Total (n = 4338)	Low risk (n = 1345)	Occasional HED (n = 1326)	Monthly HED (n = 1368)	Dependent (n = 299)	*p*
Harm to finances							
Never	3444	80.7%	94.8%	85.2%	73.7%	28.3%	<.0001
Yes once	429	10.1%	3.3%	9.8%	13.6%	25.9%	
Yes more than once	395	9.2%	1.9%	5.1%	12.7%	45.8%	
Harm to health							
Never	3550	84.2%	92.8%	86.3%	83.6%	37.7%	<.0001
Yes once	439	10.4%	5.0%	10.1%	11.5%	32.4%	
Yes more than once	226	5.4%	2.3%	3.6%	4.93%	29.9%	
Harm to work/study							
Never	3839	89.9%	95.4%	93.5%	88.7%	53.8%	<.0001
Yes once	261	6.1%	3.0%	4.4%	6.9%	24.8%	
Yes more than once	171	4.0%	1.6%	2.1%	4.4%	21.4%	
Been in a physical fight							
Never	3906	91.0%	95.9%	93.4%	89.5%	65.5%	<.0001
Yes once	288	6.7%	2.6%	4.6%	8.7%	25.6%	
Yes more than once	97	2.3%	1.4%	2.0%	1.9%	8.8%	
Harm to friendships/social life							
Never	3858	90.4%	95.8%	92.3%	90.3%	58.1%	<.0001
Yes once	280	6.6%	2.7%	5.4%	7.3%	25.6%	
Yes more than once	129	3.0%	1.5%	2.2%	2.4%	16.3%	
Stopped by the police							
Never	4020	93.8%	96.3%	94.6%	93.4%	81.0%	<.0001
Yes once	195	4. 6%	2.3%	3.6%	5.5%	14.7%	
Yes more than once	71	1.6%	1.4%	1.8%	1.1%	4.3%	
Been in an accident							
Never	3997	93.3%	96.3%	94.8%	92.2%	77.4%	<.0001
Yes once	217	5.1%	2.3%	3.7%	6.6%	16.8%	
Yes more than once	72	1.7%	1.4%	1.5%	1.2%	5.7%	
Harm to home life or marriage							
Never	3885	91.1%	95.9%	92.0%	91.1%	65.1%	<.0001
Yes once	253	5.9%	2.6%	5.6%	6.3%	21.3%	
Yes more than once	126	3.0%	1.5%	2.4%	2.6%	13.6%	

*HED* Heavy episodic drinking; Occasional HED; engaged in HED 1–11 times in the last year; Monthly HED; engaged in HED at least once a month in last year; Dependent: meets criteria for DSM-IV alcohol dependence

### Distribution of harms among drinkers in the survey

The distributions of alcohol-related harms as a percentage of all drinkers are displayed in Fig. 1 and Additional file 1: Table S1. For example, of those who experienced at least one harm to their finances in the last year, 8.4% were low-risk drinkers, 23.5% were occasional HED drinkers, 42.7% were monthly HED drinkers, and 25.4% were dependent drinkers (Fig. 1(ii)). Although individual risk of experiencing alcohol-related harm was highest among dependent drinkers, Fig. 1 illustrates that monthly HED drinkers accounted for the highest proportion of harms among drinkers (31.5–42.7%). Occasional HED drinkers accounted for between 19.8 and 27.4% of the harms, while dependent drinkers accounted for between 21.0 and 30.9% of the eight harms. Low-risk drinkers accounted for between 8.4 and 18.6% of harms.

Table 3 displays the total number of harms experienced across the four drinker types. Monthly HED drinkers accounted for the greatest proportion of the harms (34.7%). Together, occasional HED drinkers and monthly HED accounted for over half (58.7%) of the harms in the population. Dependent drinkers accounted for just over one-quarter of the harms (27.4%) and low-risk drinkers accounted for the fewest number of harms (13.9%).

**Table 3:** Total number of harms, by drinker type

	Total	Standard Error	95% CI	% share of the harms
Low risk (*n* = 1320)	652	79.5	496–807	13.9%
Occasional HED (*n* = 1276)	1122	99.8	927–1318	24.0%
Monthly HED (*n* = 1279)	1626	104.7	1421–1832	34.7%
Dependent (*n* = 272)	1281	110.6	1064–1498	27.4%
Total (*n* = 4147)	4682	188.5	4311–5051	100%

*HED*: Heavy episodic drinking; Occasional HED: engaged in HED 1–11 times in the last year; Monthly HED: engaged in HED at least once a month in last year; Dependent: meets criteria for DSM-IV alcohol dependence

### Total amount of alcohol consumed in the week prior to the survey

A total of 37,236 standard drinks were consumed by respondents in the week prior to the survey (Table 4). There was a linear association between drinker type and median number of standard drinks consumed in the last week. Dependent drinkers drank a median of 19.2 (IQR: 10.3–34.4) standard drinks in the week prior to the survey, and accounted for almost one-fifth of the alcohol consumed during the same period. Monthly HED drinkers reported drinking less (Median: 13.2; IQR: 7.8–21.5), but accounted for over half of the alcohol consumed. Together, occasional and monthly HED drinkers accounted for almost three-quarters of alcohol consumed in the week prior to the survey (71.29%).

**Table 4:** Median and total number of standard drinks consumed in week prior to survey by drinker type

	Median (IQR)	95% CI	Total number of standard drinks consumed (%)	95% CI
Low risk (*n* = 717)	3.4 (2.0–6.0)	3.05–3.79	3497 (9.39%)	3143–3854
Occasional HED (*n* = 867)	6.4 (3.8–11.1)	5.93–6.90	7453 (20.01%)	6800–8106
Monthly HED (*n* = 1137)	13.2 (7.8–21.5)	12.45–13.85	19,096 (51.28%)	17,623–20,568
Dependent (*n* = 241)	19.2 (10.3–34.4)	15.25–23.06	7189 (19.31%)	5898–8479
Total (*n* = 2997)	8.0 (4.0–15.6)	7.58–8.41	37,236 (100.00%)	35,233–39,239

*HED*: Heavy episodic drinking; Occasional HED: engaged in HED 1–11 times in the last year; Monthly HED: engaged in HED at least once a month in last year; Dependent: meets criteria for DSM-IV alcohol dependence

## Discussion

In this nationally representative study of 4338 respondents, it was found that high-risk drinkers, defined as those who met DSM-IV criteria for alcohol dependence, accounted for approximately 7% of all drinkers. This group had the greatest individual risk of experiencing all eight alcohol-related harms, but accounted for just over one-quarter (27%) of all harms experienced by survey respondents and one-fifth (19%) of all alcohol consumed in the week prior to the survey. Monthly HED drinkers represented almost one-third of all drinkers, consumed 51% of the alcohol and accounted for just over one-third (35%) of the harms. Monthly and occasional HED drinkers combined accounted for 62% of all drinkers, consumed 70% of the alcohol and accounted for 59% of the harms. Low-risk drinkers, defined as those who did not engage in HED in the last year and did not meet the DSM-IV criteria for alcohol dependence, accounted for 31% of all drinkers, 9% of alcohol consumed in the last week and 14% of the harms.

These findings provide evidence for both the prevention paradox and the “second order” prevention paradox in the Irish population when the high-risk group is defined in terms of alcohol dependence. In line with previous findings on the prevention paradox, the majority of harms occurred to low-risk or moderate-risk drinkers, although dependent drinkers had a much higher individual risk of experiencing alcohol-related harms [6, 8, 11, 14, 16]. In support of the “second order” prevention paradox, the majority of the harms were accounted for by people who engaged in HED [9, 10, 12–15]. This is consistent with findings from other countries. For example, in a study of adolescent drinking in 23 European countries, Danielsson et al. [6] reported that in almost all countries, heavy episodic drinkers in the bottom 90% of consumers by volume accounted for the majority of alcohol-related harms. Caetano and colleagues [9, 10] reported similar findings in a population-based sample of adults in Brazil and amongst a sample of Hispanic American adults. As with our data, these findings imply that most alcohol-related-harms are due to periods of acute intoxication, and because these occasions are most numerous among low and moderate drinkers, they account for the majority of alcohol-related harms [6, 13, 14].

Despite alcohol dependence being associated with the most detrimental alcohol-related health and social consequences [3, 4, 24], few studies have included dependence as a drinking pattern when examining the validity of the prevention paradox. In an exception to this case, Dawson [25] investigated the association between alcohol consumption, incidence of drink-driving, and alcohol dependence amongst a population-based sample of US adults. Dawson [25] reported that approximately 10% of the sample met DSM-IV criteria for alcohol dependence and accounted for the majority (57%) of incidents of drink driving in the last 12 months. However, our findings indicate that the majority of harms in the Irish population were not accounted for by dependent drinkers. This is possibly due to the high prevalence of HED among non-dependent drinkers in this study. The majority of non-dependent drinkers had engaged in at least one episode of HED in the last year, considerably higher than findings from other countries [10, 12, 14] indicating that this pattern of consumption is the norm in Ireland. Of note, low-risk drinkers accounted for a considerable minority of the harms (14%), indicating that low-risk does not equate to “no risk” when it comes to alcohol consumption.

### Strengths and limitations

This study had a number of key strengths. Firstly, the large sample size and stratified clustering approach to sampling meant that the survey was based on a representative sample and so the results can be generalised to the whole Irish population. HED and alcohol dependence were measured using validated and reliable questionnaires, strengthening the validity and reliability of the findings. Defining the high-risk group in terms of alcohol dependence, and examining the validity of the prevention paradox in terms of patterns of drinking rather than volume of alcohol consumed, was a novel way of studying the prevention paradox.

Despite these strengths, the findings of this study must be interpreted in the light of several methodological limitations. Although our findings are based on a nationally representative sample, response bias may have been an issue. General population surveys often fail to capture the heaviest drinkers [26]. Self-report bias is a common feature of alcohol consumption surveys and may result in under-estimation of alcohol consumption [27, 28]. Previous analyses demonstrated that the NADS survey accounted for just 39% of recorded alcohol sales in the year it was carried out [22]. This is despite explaining the concept of a standard drink in detail to respondents and providing corresponding visual aids. Furthermore, alcohol-related harms in this study were self-reported and so may also be subject to self-report bias.

### Policy implications

Research indicates that the most effective approaches to reducing alcohol consumption are those which target the entire population, such as increasing the price and reducing the availability of alcohol [4]. In Ireland, the Public Health (Alcohol) Act (2018) [21] has recently (October, 2018) been passed and will introduce a number of population-based strategies to reduce alcohol consumption, including a minimum unit price for alcohol sales and restrictions on advertising and marketing. This policy represents one of the most progressive alcohol policies in the world. It was fiercely contested by the alcohol industry and the interval between the publication of the Bill and the passage of the Act at 3 years was the longest ever in Ireland. None of the provisions in the Act have yet been implemented. The findings in this study demonstrate that alcohol-related harms in Ireland are distributed across the population, providing support for a population-based strategy to reducing alcohol consumption. The findings thus indicate a need for the rapid implementation of measures outlined in the Public Health (Alcohol) Act (2018).

### Future research

It is important to note that factors other than those included in the study may contribute to alcohol-related harms. For example, a population-based study of drinking habits in Finland [29] demonstrated that after holding drinking patterns constant, drinking context was associated with different types of harms. Future research could examine the association between drinking context and alcohol-related harms in Ireland. Additionally, the harms included in the study were self-reported; in other countries, the prevention paradox has been found to be valid across more serious harms such as hospitalisations and alcohol-related deaths [16]. Further research could explore if these findings could be replicated in the Irish context.

## Conclusions

The majority of alcohol-related harms in Ireland occurred among drinkers who engage in heavy episodic drinking. Almost two-thirds of drinkers reported engaging in heavy episodic drinking in the last 12 months, indicating that hazardous patterns of drinking are the norm in Ireland. Given the distribution of alcohol-related harm, a population-based approach to reducing alcohol consumption such as that outlined in the Public Health (Alcohol) Act (2018) is therefore the most appropriate policy strategy to reduce alcohol-related harm in Ireland.

## Supplementary information

**Additional file 1:** **Table S1.** Distribution of harms across drinker type. (DOCX 14 kb)

## Data Availability

The dataset used and/or analysed for the purpose of this study are available from the corresponding author on reasonable request.

## Abbreviations

CAPI: Computer Assisted Personal Interview
DSM-IV: Diagnostic and Statistical Manual of Mental Disorders, 4th Edition
HED: Heavy episodic drinking
NADS: National Alcohol Dairy Survey
OECD: Organisation for Economic Co-operation and Development (OECD)
SMART: Standardized Measurement of Alcohol-Related Troubles

## References

[1] Griswold MG, Fullman N, Hawley C, Arian N, Zimsen SR, Tymeson HD (2018). Alcohol use and burden for 195 countries and territories, 1990–2016: a systematic analysis for the global burden of disease study 2016. Lancet.

[2] Room R, Graham K, Rehm J, Jernigan D, Monteiro M (2003). Drinking and its burden in a global perspective: policy considerations and options. Eur Addict Res.

[3] Rehm J, Mathers C, Popova S, Thavorncharoensap M, Teerawattananon Y, Patra J (2009). Global burden of disease and injury and economic cost attributable to alcohol use and alcohol-use disorders. Lancet.

[4] Babor T, Caetano R, Casswell S, Edwards G, Giesbrecht N, Graham K (2010). Alcohol: no ordinary commodity: research and public policy.

[5] Manthey J, Shield KD, Rylett M, Hasan OS, Probst C, Rehm J (2019). Global alcohol exposure between 1990 and 2017 and forecasts until 2030: a modelling study. Lancet.

[6] Danielsson AK, Wennberg P, Hibell B, Romelsjö A (2012). Alcohol use, heavy episodic drinking and subsequent problems among adolescents in 23 European countries: does the prevention paradox apply?. Addiction.

[7] McCambridge J, Mialon M, Hawkins B (2018). Alcohol industry involvement in policymaking: a systematic review. Addiction.

[8] Kreitman N (1986). Alcohol consumption and the preventive paradox. Br J Addict.

[9] Caetano R, Mills BA (2011). The Hispanic Americans baseline alcohol survey (HABLAS): is the “prevention paradox” applicable to alcohol problems across Hispanic national groups?. Alcohol Clin Exp Res.

[10] Caetano R, Mills B, Pinsky I, Zaleski M, Laranjeira R (2012). The distribution of alcohol consumption and the prevention paradox in Brazil. Addiction.

[11] Rossow I, Romelsjö A (2006). The extent of the ‘prevention paradox’in alcohol problems as a function of population drinking patterns. Addiction.

[12] Kraus L, Baumeister SE, Pabst A, Orth B (2009). Association of average daily alcohol consumption, binge drinking and alcohol-related social problems: results from the German epidemiological surveys of substance abuse. Alcohol Alcoholism.

[13] Stockwell T, Hawks D, Lang E, Rydon P (1996). Unravelling the preventive paradox for acute alcohol problems. Drug Alcohol Review.

[14] Gmel G, Klingemann S, Müller R, Brenner D (2001). Revising the preventive paradox: the Swiss case. Addiction.

[15] Skog OJ (1999). The prevention paradox revisited. Addiction.

[16] Poikolainen K, Paljärvi T, Mäkelä P (2007). Alcohol and the preventive paradox: serious harms and drinking patterns. Addiction.

[17] World Health Organization. Global status report on alcohol and health. Geneva: 2014.

[18] Department of Health I. Healthy Ireland survey 2018: summary of findings. Dublin: 2018.

[19] Organisation for Economic Co-operation and Development. Alcohol consumption 2019 [June 2019]. Available from: https://data.oecd.org/healthrisk/alcohol-consumption.htm. Accessed 07 Sept 2019.

[20] Long JM, D. (2014). Alcohol consumption in Ireland 2013: analysis of a national alcohol diary survey.

[21] Government of Ireland. Public Health Alcohol Act. 2018.

[22] Kessler RC, Ustun TB (2004). The world mental health (WMH) survey initiative version of the World Health Organization (WHO) composite international diagnostic interview (CIDI). Int J Methods Psychiatr Res.

[23] Moskalewicz J, Sieroslawski J (2010). Drinking population surveys–guidance document for standardized approach.

[24] Rehm J, Anderson P, Barry J, Dimitrov P, Elekes Z, Feijao F (2015). Prevalence of and potential influencing factors for alcohol dependence in Europe. Eur Addict Res.

[25] Dawson DA (1999). Alternative definitions of high risk for impaired driving: the overlap of high volume, frequent heavy drinking and alcohol dependence. Drug Alcohol Depend.

[26] Caetano R (2001). Non-response in alcohol and drug surveys: a research topic in need of further attention. Addiction.

[27] Lahaut VM, Jansen HA, Van de Mheen D, Garretsen HF (2002). Non-response bias in a sample survey on alcohol consumption. Alcohol Alcohol.

[28] Meiklejohn J, Connor J, Kypri K (2012). The effect of low survey response rates on estimates of alcohol consumption in a general population survey. PLoS One.

[29] Mäkelä P, Mustonen H, Lintonen T (2016). Connection between drinking context choices and self-reported alcohol-related social harm: results from the Finnish drinking habit survey 2008. Drug Alcohol Rev.

