# Supplementary material for: Drinking patterns and the distribution of alcohol-related harms in Ireland: evidence for the prevention paradox
Source: BMC Public Health. 2019 Oct 22;19:1323. doi: 10.1186/s12889-019-7666-4 (PMC6805445; doi:10.1186/s12889-019-7666-4)
Supplement: Supplementary file 1 — Additional file 1: Table S1. Distribution of harms across drinker type. (DOCX 14 kb) [file 12889_2019_7666_MOESM1_ESM.docx]

**Additional file 1: Table S1. Distribution of harms across drinker type.**

|  | **n** | **Low risk (n=1345)** | **Occasional HED (n=1326)** | **Monthly HED (n=1368)** | **Dependent (n=299)** | **p** |
| --- | --- | --- | --- | --- | --- | --- |
| **Harm to finances** | 823 | 8.4% | 23.5% | 42.7% | 25.4% | <.0001 |
| **Harm to health** | 665 | 14.4% | 26.6% | 32.4% | 26.7% | <.0001 |
| **Harm to work/study** | 432 | 14.2% | 19.8% | 35.2% | 30.9% | <.0001 |
| **Been in a physical fight** | 384 | 13.9% | 22.7% | 37.1% | 26.3% | <.0001 |
| **Harm to friendships/social life** | 409 | 13.7% | 24.5% | 31.9% | 29.9% | <.0001 |
| **Stopped by the police** | 266 | 18.6% | 26.8% | 33.6% | 21.0% | <.0001 |
| **Been in an accident** | 288 | 17.0% | 23.8% | 36.5% | 22.7% | <.0001 |
| **Harm to home life or marriage** | 379 | 14.4% | 27.4% | 31.5% | 26.7% | <.0001 |

*HED: Heavy episodic drinking; Occasional HED; engaged in HED 1-11 times in the last year; Monthly HED; engaged in HED at least once a month in last year; Dependent: meets criteria for DSM-IV alcohol dependence*
